# Are Proteinopathy and Oxidative Stress Two Sides of the Same Coin?

**DOI:** 10.3390/cells8010059

**Published:** 2019-01-16

**Authors:** Nihar J. Mehta, Praneet Kaur Marwah, David Njus

**Affiliations:** 1 Radiation Oncology Branch, Center for Cancer Research, National Cancer Institute, National Institutes of Health, Bethesda, MD 20892, USA; nihar.mehta@nih.gov; 2 Department of Biological Sciences, Wayne State University, Detroit, MI 48202, USA; praneet.marwah@wayne.edu

**Keywords:** autophagy, cysteinyl-dopamine, hypochlorite, oxidative stress, Parkinson’s disease, redox cycling

## Abstract

Parkinson’s disease, like other neurodegenerative diseases, exhibits two common features: Proteinopathy and oxidative stress, leading to protein aggregation and mitochondrial damage respectively. Because both protein aggregates and dysfunctional mitochondria are eliminated by autophagy, we suggest that inadequate clearance may couple the two phenomena. If a neuron’s autophagy machinery is overwhelmed, whether by excessive oxidative stress or by excessive protein aggregation, protein aggregates and dysfunctional mitochondria will both accumulate. Parkinson’s disease may provide a unique window into this because there is evidence that both sides contribute. Mutations amplifying the aggregation of α-synuclein are associated with Parkinson’s disease. Likewise, mutations in Parkin and PINK1, proteins involved in mitophagy, suggest that impaired mitochondrial clearance is also a contributing factor. Many have suggested that dopamine oxidation products lead to oxidative stress accounting for the dopaminergic selectivity of the disease. We have presented evidence for the specific involvement of hypochlorite-oxidized cysteinyl-dopamine (HOCD), a redox-cycling benzothiazine derivative. While toxins like 6-hydroxydopamine and 1-methyl-4-phenyl pyridinium (MPP+) have been used to study mitochondrial involvement in Parkinson’s disease, HOCD may provide a more physiologically relevant approach. Understanding the role of mitochondrial dysfunction and oxidative stress in Parkinson’s disease and their relation to α-synuclein proteinopathy is important to gain a full picture of the cause, especially for the great majority of cases which are idiopathic.

## 1. Introduction

Neurodegenerative diseases, such as Alzheimer’s, Parkinson’s, Huntington’s, and amyotrophic lateral sclerosis (ALS), are commonly associated with both protein aggregation and oxidative stress. The protein deposits are clearly visible and have attracted considerable attention. Oxidative stress has been much more elusive, and cellular processes contributing to it have been vaguely defined. The dopaminergic neurons that die in Parkinson’s disease are unusually prone to mutations in mitochondrial quality-control factors, such as Parkin and PINK1 [1,2], to mitochondrial toxins like rotenone [3], and to dopamine oxidation products [4,5,6,7,8,9,10,11]. As a consequence, Parkinson’s disease may offer a unique window into the role of oxidative stress in neurodegenerative diseases generally.

**Figure 1:**
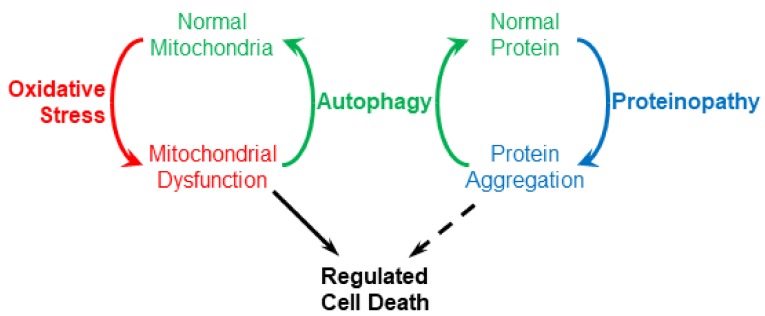
Autophagy (specifically macroautophagy) couples proteinopathy and oxidative stress.

While oxidative stress and proteinopathy are usually studied separately, an integrated perspective may help synthesize what we know about neurodegenerative diseases. Oxidative stress and proteinopathy are linked by autophagy, which is a normal cellular mechanism for clearing both dysfunctional mitochondria and aggregated proteins (Figure 1). This implies that a problem with autophagy will result in accumulation of both protein aggregates and dysfunctional mitochondria, consistent with the coincident occurrence of proteinopathy and oxidative stress. A further implication is that autophagy overload may be caused by either excessive protein aggregation or extreme oxidative stress. In neurodegenerative diseases, what might cause the normal cycling of material by autophagy to spiral out of control leading to the death of neurons?

In the context of Parkinson’s disease (PD), the unique vulnerability of dopamine neurons to oxidative stress has received considerable attention. Much of that has focused on dopamine, the defining component of dopaminergic neurons, and on potentially toxic products formed by its oxidation [6,7,8,9,10,11]. Although candidate toxins have been elusive, we recently described hypochlorite-oxidized cysteinyl-dopamine (HOCD), a cytotoxin formed by exposing cysteinyl-dopamine to hypochlorite [4]. Hypochlorite is itself a contributor to oxidative stress, and the enzyme that produces it, myeloperoxidase, is induced by low concentrations of rotenone and by HOCD itself. HOCD is a potent redox cycler and may increase oxidative stress and accelerate the formation of dysfunctional mitochondria. Therefore, HOCD along with α-synuclein aggregation may contribute to an excessive and unsustainable demand for autophagy, ultimately triggering regulated cell death. To put this in a broader context, we will begin with brief discussions of autophagy/proteinopathy and mitophagy/oxidative stress and then consider how HOCD may contribute to the selective death of dopaminergic neurons.

## 2. Proteinopathy and Autophagy

It is well known that proteinopathies or formation of protein deposits are a common feature of neurodegenerative diseases. The plaques and tangles formed by deposits of amyloid β and tau proteins are hallmarks of Alzheimer’s disease. The cytoplasmic inclusion bodies called Lewy bodies containing aggregates of α-synuclein are characteristic of Parkinson’s disease. Aggregation of polyglutamate variants of huntingtin is a cause of Huntington’s disease. And aggregation of TDP-43 and/or superoxide dismutase 1 is observed in ALS. Mutations in these proteins are associated with familial forms of these diseases arguing that the protein aggregates can contribute to neurodegeneration. Accordingly, much work has focused on the toxicity of specific protein aggregates. 

In all proteinopathies, it can be debated whether lethality is attributable to intrinsic toxicity of the protein or to the aggregated state itself. In either case, however, the problem lies in the accumulation of the protein. Evidence supports the view that protein aggregates are removed by autophagy [12,13,14,15,16,17,18,19,20]. Moreover, there is good evidence that enhancing autophagy is beneficial for treating neurodegenerative diseases [13,14,15,17,21]. Therefore, we can imagine that protein aggregation leads to autophagy, and problems arise when the capacity of the neuron to clear these deposits by autophagy is exceeded. 

Following common practice, we will use the term autophagy to refer specifically to macroautophagy. Autophagy is a highly selective process in which damaged/aggregated proteins or damaged organelles are marked by ubiquitin for elimination. When mitochondria are the target, the process is commonly called mitophagy. In either case, the process begins with the formation of a double-membrane structure called the isolation membrane or phagophore. It wraps around the material to be eliminated forming a closed structure called the autophagosome. Finally, lysosomes fuse with the autophagosome creating an autolysosome, and lysosomal hydrolases then degrade the material inside. During the process, a cytosolic protein, microtubule-associated protein 1A/1B light chain 3B (LC3-I) is recruited to the autophagosomal membrane where it is lipidated with phosphatidylethanolamine to form LC3-phosphatidylethanolamine conjugate (LC3-II). LC3-II, therefore, is useful as a marker for autophagosomes. For reviews of autophagy see References [22,23]. 

Autophagy/mitophagy is a receptor-mediated process; specific receptors including p62 and optineurin simultaneously interact with the cargo and LC3-II on the isolation membrane, thus recruiting the damaged protein/organelle to the autophagosome. These receptors have an LC3-interacting region (LIR) and a ubiquitin-binding domain (UBD) that binds to ubiquitin chains on the target protein/organelle. Ubiquitin and p62 seem to be more commonly associated with protein deposits. For example, p62 has been reported in α-synuclein deposits in neuron-specific autophagy-deficient mice [24]. On the other hand, in mitophagy, optineurin seems to be the favored receptor [25,26].

With specific relevance to Parkinson’s disease, both glucocerebrosidase and leucine-rich repeat kinase 2 seem to be needed for proper functioning of the autophagy/lysosome pathway [27,28,29]. Mutations in genes for both proteins (*GBA1* and *LRRK2*) are associated with familial cases of Parkinson’s disease.

What happens when a cell cannot keep pace with the accumulation of cellular debris? An emerging view is that autophagy is just the normal first response. If a cell is so dysfunctional that its autophagic machinery cannot keep up, that cell is eliminated by regulated cell death as a last resort [22,23]. Historically, regulated cell death was more or less synonymous with apoptosis. As new pathways of regulated cell death were discovered, however, new nomenclature was required. The Nomenclature Committee on Cell Death 2018 has defined twelve major cell death subroutines [30]. Two, intrinsic apoptosis and parthanatos, are of interest here.

Intrinsic apoptosis has been studied extensively. It involves mitochondrial outer membrane permeabilization (MOMP), followed by release of cytochrome c and other factors into the cytosol. This activates a caspase cascade ultimately resulting in cell death. A multitude of regulatory proteins are also involved. Given the detail with which intrinsic apoptosis is understood, it is apparent that it and autophagy are mutually inhibitory [22,23]. Autophagy inhibits apoptosis by eliminating dysfunctional mitochondria that might otherwise trigger the intrinsic apoptotic pathway. Autophagy also degrades components of the apoptosis cascade including caspases and the BH3-only protein NOXA. Apoptosis, on the other hand, inhibits autophagy because apoptotic caspases degrade Beclin-1, a protein involved in expansion of the isolation membrane in autophagy [31]. Thus, the two processes are coordinated so they do not occur concurrently. Autophagy is the normal response to clear unwanted material; if that fails, death by apoptosis may occur.

Parthanatos is a mode of regulated cell death involving hyperactivation of poly(ADP-ribose)polymerase 1 (PARP1) and occurs in response to DNA damage and oxidative stress [30]. Hyperactivation of PARP1 results in the production of poly(ADP-ribose) which binds to apoptosis inducing factor (AIF), causing its release from mitochondria and translocation to the nucleus where it promotes DNA fragmentation. Significantly, protein aggregates, including α-synuclein [32] and amyloid β [33] seem to trigger hyperactivation of PARP1. The dopaminergic neurotoxins MPTP and 6-hydroxydopamine also affect PARP1 [34,35], and we have found that HOCD causes cleavage of PARP1 in PC12 cells [36]. All of this marks parthanatos as a prime contender for the cause of neuronal death in neurodegenerative disease. The interplay between autophagy and parthanatos is not yet clear. One would presume, as with intrinsic apoptosis, that parthanatos is the last resort after autophagy and other protective mechanisms fail. How parthanatos interacts with autophagy and its role in neuronal death are clearly significant questions. 

## 3. Mitochondrial Dysfunction and Mitophagy

Several lines of evidence show that mitochondrial dysfunction plays a pivotal role in the pathogenesis of Parkinson’s disease and other neurodegenerative disorders [37,38,39,40]. Disruption of electron flow through the respiratory chain as well as other metabolic reactions in the mitochondrion can produce reactive oxygen (ROS) and reactive nitrogen species (RNS), thus contributing to oxidative stress [41]. This ROS/RNS generation can cause irreversible damage to DNA, lipids, and proteins. This is especially significant in mitochondria, which lack many of the repair mechanisms available in the cytosol and nucleus and which are prone to oxidation because of the relatively high pH in the matrix. Mitochondrial damage, especially inhibition of complex I and other enzymes involved in the respiratory chain, has been suggested as one of the fundamental causes of Parkinson’s disease [42,43]. In this connection, Complex I inhibition by rotenone increases ROS production [44], and low concentrations of rotenone selectively kill dopaminergic neurons [3]. 

To prevent the accumulation of damaged, ROS-producing mitochondria, elimination of dysfunctional mitochondria is essential. Mitophagy uses the machinery of autophagy for this selective degradation of senescent and damaged mitochondria, in order to maintain a healthy mitochondrial pool. Several types of mitophagy have been described differing in the mechanism by which mitochondria are engulfed by the autophagosome prior to degradation in the lysosomes. The most well characterized is mitophagy mediated by two proteins—PTEN-induced kinase 1 or PINK1 (a serine/threonine kinase) and Parkin (an E3-ubiquitin ligase). In healthy and polarized mitochondria, PINK1 is imported to the inner membrane where it is cleaved by mitochondrial proteases such as mitochondrial processing peptidase (MPP) and presenilin-associated rhomboid-like protein (PARL) [45,46,47]. However, mitochondrial inner membrane depolarization, a sign of a damaged mitochondrion, stabilizes PINK1 [48], which then phosphorylates serine 65 of ubiquitin and the N-terminal ubiquitin-like domain of Parkin [49,50,51]. Phosphorylation of parkin activates its E3 ubiquitin ligase activity, resulting in ubiquitination of mitochondrial proteins, targeting them for degradation by autophagy. 

Genetic studies have revealed that mutation in the genes *PRKN* and *PARK6*, which encode for Parkin and PINK1 respectively, are linked to autosomal recessive cases of early-onset or juvenile forms of PD [1,2]. In addition, the *PARK7* gene, also linked to autosomal recessive early-onset cases of PD, encodes for the protein deglycase DJ-1, which also promotes autophagy and maintenance of mitochondrial function [52]. The identification of these mutations in familial forms of PD clearly suggests that impaired mitochondrial turnover is a key feature in the pathogenesis of PD. Moreover, mitophagy is not only impaired in PD, but accumulating evidence suggests that dysfunctional autophagy/mitophagy is also manifested in other neurodegenerative disorders such as Alzheimer’s disease [53,54], Huntington’s disease [14,55], and ALS [25,56,57]. 

As Parkin/PINK1-mediated mitophagy depends on the loss of mitochondrial inner membrane potential, it is not surprising that mitophagy is initiated by a variety of mitochondrial toxins. These include the protonophore FCCP, the respiratory chain inhibitor antimycin, and the ATP synthase inhibitor oligomycin. Others include the dopaminergic toxins 6-hydroxydopamine and 1-methyl-4-phenylpyridinium (MPP+) and the pesticide rotenone [58]. 

Pioneering work by the Greenamyre group established that chronic, systemic exposure to rotenone can produce two major hallmarks of Parkinson’s disease: Selective dopaminergic neuron degeneration and α-synuclein accumulation in cytoplasmic inclusions resembling Lewy bodies [3]. Because rotenone is an inhibitor of Complex I of the mitochondrial respiratory chain, this has been considered evidence for the involvement of mitochondrial dysfunction in PD. Rotenone treatment has other effects as well, however. Especially interesting is a link between rotenone and myeloperoxidase expression. Chang et al. [59] demonstrated that rotenone-induced neurotoxicity can be mitigated by modulating myeloperoxidase levels. Moreover, we have reported that rotenone increases the expression of myeloperoxidase in PC12 cells which, by forming hypochlorite, leads to the formation of a toxic redox cycler, HOCD [4]. HOCD formation is exclusive to dopaminergic neurons since it is formed by hypochlorite-mediated oxidation of cysteinyl-dopamine, a product of dopamine oxidation. Interestingly, myeloperoxidase is a lysosomal enzyme, and this may account for its upregulation by agents such as rotenone that promote autophagy/mitophagy.

## 4. Dopamine Oxidation and HOCD

**Figure 2:**
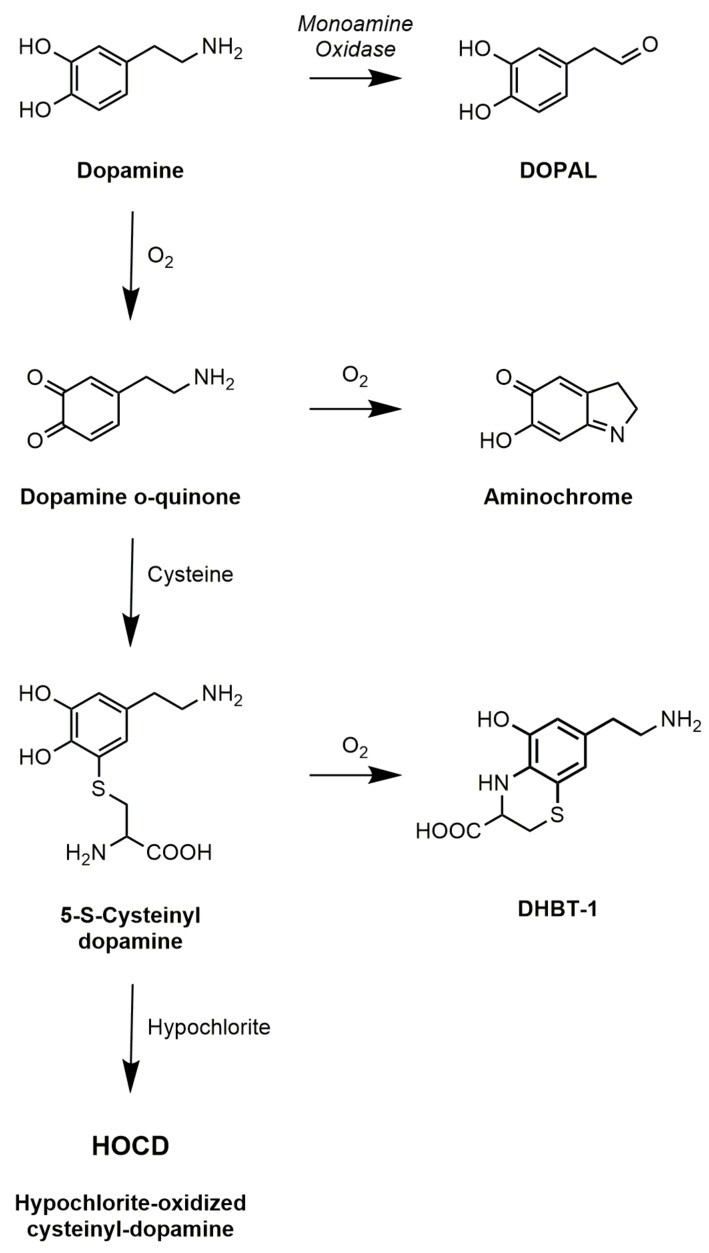
Products of dopamine oxidation.

Following the discovery that Parkinson’s disease is associated with the extensive loss of dopamine neurons in the substantia nigra, there has been considerable speculation that dopamine oxidation leads to the formation of toxic products. Some of this has focused on normal products of dopamine metabolism, in particular 3,4-dihydroxyphenylacetaldehyde (DOPAL), which is the immediate product of the enzyme monoamine oxidase (Figure 2). The aldehyde is normally converted to 3,4-dihydroxyphenylacetic acid (DOPAC) by aldehyde dehydrogenase. The aldehyde, however, can conjugate with amines in proteins altering the activity of those proteins [60], and inhibition of aldehyde dehydrogenase does lead to increased toxicity of dopamine [61]. 

Most attention, however, has focused on the non-enzymatic oxidation of dopamine. Using induced pluripotent stem cells from genetic and sporadic PD patients, Burbulla et al. [9] found that elevated mitochondrial oxidative stress levels can trigger accumulation of dopamine oxidation adducts which, together with mutation in DJ-1, initiates a toxic cascade resulting in α-synuclein accumulation. Dopamine undergoes spontaneous auto-oxidation to form the dopamine quinone. This is accelerated in the presence of metal ions such as iron or copper, so these would be expected to exacerbate effects of dopamine oxidation. The dopamine quinone itself has been cited as a toxin [10], but it is unstable and either cyclizes to form aminochrome or conjugates with thiols to form products such as 5-S-cysteinyl-dopamine (Figure 2). Aminochrome continues to receive attention [11], but it is neither a very potent neurotoxin nor the main product of dopamine oxidation in vivo. The predominant product in vivo, given the pervasive presence of cysteine, is cysteinyl-dopamine. Carlsson and his colleagues [62] detected cysteinyl-dopamine in the cerebrospinal fluid of PD patients, in dopamine-rich regions of the brain such as the caudate nucleus, putamen, globus pallidus, and substantia nigra, and in neuromelanin. Cysteinyl-dopamine has been reported to kill neuronal cells [6,8], but it is uncertain whether it is cytotoxic itself or metabolizes to toxic products. Dryhurst and colleagues [63] identified many products formed by the oxidation of dopamine in the presence of cysteine. They found that DHBT-1 (7-(2-aminoethyl)-3,4-dihydro-5-hydroxy-2H-1,4-benzothiazine-3-carboxylic acid) is the principal product formed by air oxidation of cysteinyl-dopamine. It inhibits mitochondrial Complex I but is only weakly cytotoxic requiring millimolar concentrations. Treatment of cells with cysteinyl-dopamine can result in oxidative damage, a rise in intracellular calcium, and ultimately apoptosis. Recently, Vauzour et al. [8] attributed its toxicity to combined effects of cysteinyl-dopamine itself and DHBT-1.

Oxidized dopamine and cysteinyl-dopamine also polymerize to form neuromelanin. Neuromelanin is a stable substance, and it may confer protection by acting as a sink for oxidized dopamine products and by chelating iron [64]. However, neuromelanin may also contribute to increased susceptibility of melanized neurons due to accumulation of increased loads of iron and toxic metabolites. Moreover, microglia activation by neuromelanin released from degenerating neurons can further contribute to neurodegeneration. 

**Figure 3:**
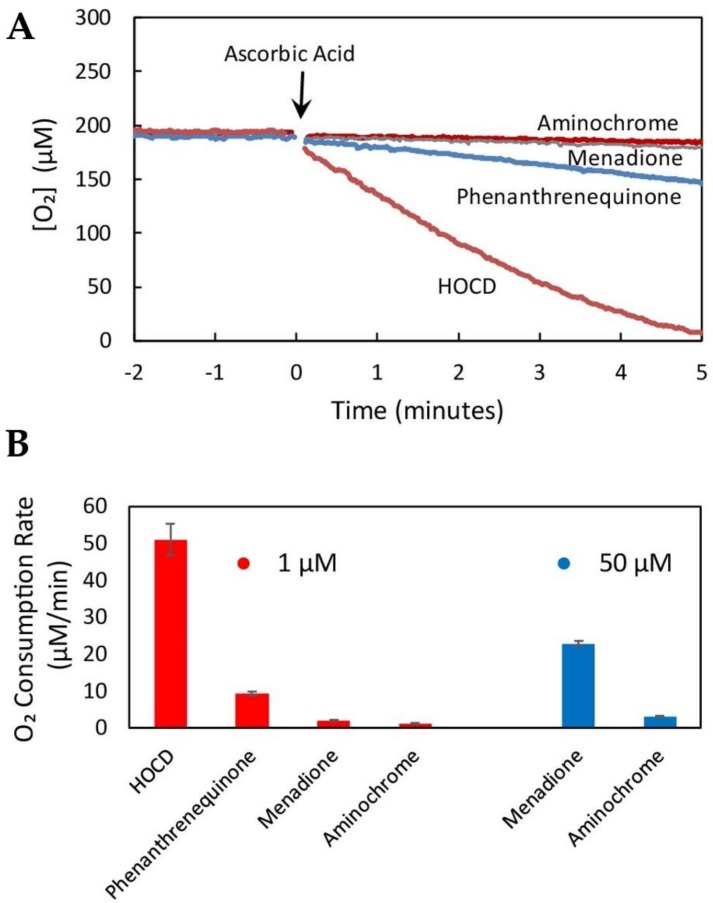
Hypochlorite-oxidized cysteinyl-dopamine (HOCD) undergoes extremely rapid redox cycling. (**A**) Oxygen consumption mediated by 1 µM concentrations of the indicated redox cyclers following addition of 2.5 mM ascorbic acid (arrow). Oxygen consumption was measured in aqueous solution (0.2 M potassium phosphate, 1 µM EDTA, pH 7.4) at 37 °C. (**B**) Comparison of redox cycling rates (initial slopes of plots shown in 3A) by 1 µM concentrations of redox cyclers (red bars) or 50 µM concentrations (blue bars). Averages (± standard deviation) of three replicate samples are shown (authors’ unpublished data).

Rather than examine toxicity of specific products, we chose to approach the problem by looking for an activity: Redox cycling. The process of redox cycling involves alternating reduction and oxidation reactions continuing until either molecular oxygen or reducing equivalents are exhausted. This leads to the proliferation of a variety of reactive oxygen species including superoxide and hydrogen peroxide, so redox cycling agents can induce oxidative stress and mitochondrial dysfunction. Because cysteinyl-dopamine is the primary product of dopamine oxidation in vivo, we chose to seek redox cycling products formed from cysteinyl-dopamine. We [4] discovered that treatment of cysteinyl-dopamine with hypochlorite yields a product with very high redox cycling activity (Figure 3), and we refer to this product as HOCD (hypochlorite-oxidized cysteinyl-dopamine).

Using PC12 cells, we confirmed that cysteinyl-dopamine is toxic. However, HOCD is toxic at lower concentrations. Moreover, two lines of evidence suggest that the toxicity of cysteinyl-dopamine depends on its conversion to HOCD. First, including taurine in the medium protects PC12 cells against cysteinyl-dopamine but not against HOCD. Taurine scavenges hypochlorite and blocks the hypochlorite-dependent conversion of cysteinyl-dopamine into HOCD in vitro. Thus, it is likely that taurine also prevents this conversion in vivo, thereby protecting cells against cysteinyl-dopamine but not against HOCD.

The second line of evidence is that rotenone potentiates the toxicity of cysteinyl-dopamine but not of HOCD. Consistent with reports of others, we found that low concentrations of rotenone increase myeloperoxidase expression in PC12 cells. The resulting increase in hypochlorite due to increased myeloperoxidase activity would be expected to yield more effective conversion of cysteinyl-dopamine to HOCD, thereby increasing toxicity of cysteinyl-dopamine but not of HOCD. In this connection, Gellhaar et al. [65] found that brain regions affected in PD patients show significant increases in myeloperoxidase immunoreactivity, providing further evidence that myeloperoxidase may mediate the selective vulnerability of dopaminergic neurons to oxidative stress.

We have succeeded in scaling up the synthesis of HOCD and have purified the redox cycling product by chromatography through Dowex 50Wx8. It appears to be a 1,4-benzothiazine with the dopamine oxygens on the 7 and 8 carbons. This puts an O para to the N and allows the compound to undergo facile oxidation/reduction. In fact, HOCD redox cycles faster than any compound we have tested using our standard redox cycling assay (measured as rate of oxygen consumption in 0.2 M potassium phosphate, 1 µM EDTA, pH 7.4 in the presence of 2.5 mM ascorbic acid at 37 °C). HOCD is two orders of magnitude faster than menadione and three orders of magnitude faster than aminochrome (Figure 3).

**Figure 4:**
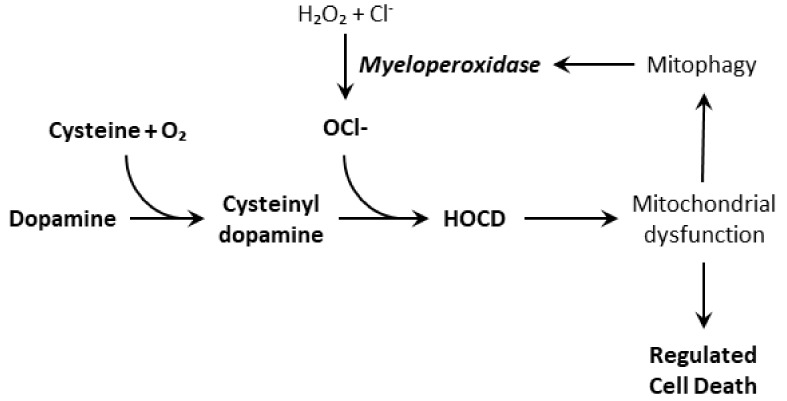
Formation of hypochlorite-oxidized cysteinyl-dopamine from dopamine and its self-enhancement by increasing myeloperoxidase expression.

Because of this fast redox-cycling, we suspect that HOCD contributes to oxidative stress and damage to mitochondria. This is suggested by the fact that other redox active compounds, such as 6-hydroxydopamine and aminochrome, do the same. Moreover, HOCD causes a rapid increase in superoxide levels in PC12 cells as observed using the fluorescent mitochondrial superoxide indicator MitoSOX Red [4]. Finally, HOCD causes an increase in expression and activity of the lysosomal enzyme myeloperoxidase, perhaps by increasing mitophagy. A consequence of the upregulation of myeloperoxidase is increased hypochlorite production, which should increase HOCD formation. This self-reinforcing feedback should cause oxidative stress to spiral out of control (Figure 4). For neurodegenerative diseases, this self-reinforcing feedback is especially significant. Something must change a manageable situation into one that accelerates uncontrollably. Our results with PC12 cells indicate that neuronal myeloperoxidase may produce enough hypochlorite to convert cysteinyl-dopamine to HOCD, but it is also possible that microglial myeloperoxidase plays a significant or even dominant role in vivo. Thus, inflammation stimulated by α-synuclein deposits may also promote HOCD formation.

A question that is important but difficult to answer now is whether HOCD reaches toxic concentrations under physiological conditions. In most cases, the answer is clearly no, because most people do not suffer from Parkinson’s disease. But toxicity depends on context. How robust or how compromised are cellular protective mechanisms including autophagy/mitophagy? To what extent are neurons under assault by other factors such as protein aggregation or other sources of oxidative stress? And do these factors interact amplifying the total stress on the neuron? Then, HOCD may be the extra burden that makes dopaminergic neurons uniquely vulnerable in Parkinson’s disease.

## 5. Conclusions

In summary, we view Parkinson’s disease as a problem of autophagy overload caused by the combined accumulation of dysfunctional mitochondria and aggregated α-synuclein. Under normal circumstances, mitochondrial degeneration and protein aggregation occur at rates slow enough for autophagy to maintain homeostasis. Under pathological conditions, however, something causes these to accelerate out of control. The two factors may contribute differently in different patients. In familial cases involving mutations in α-synuclein or duplication or triplication of the *SNCA* gene, excessive protein aggregation is likely the dominant contributor. In cases involving mutations in Parkin or PINK1, inadequate mitophagy is the obvious culprit. In the great majority of idiopathic cases, environmental toxins or dopamine oxidation products such as HOCD may make a significant contribution. Protein aggregation and oxidative stress may also interact. Oxidative stress-induced damage of α-synuclein can enhance its oligomerization and aggregation [66,67]. Moreover, α-synuclein aggregation may exacerbate oxidative stress. Therefore, proteinopathy and oxidative stress may be synergistic, not simply additive, mutually escalating the rates at which they occur. This kind of positive feedback is essential to push the normal clearance of material by autophagy out of control. Then, if overwhelming autophagy is the signal for regulated cell death in Parkinson’s and other neurodegenerative diseases, proteinopathy and oxidative stress must be considered as a whole; they are two sides of the same coin.

## References

[1] Kitada T., Asakawa S., Hattori N., Matsumine H., Yamamura Y., Minoshima S., Yokochi M., Mizuno Y., Shimizu N. (1998). Mutations in the parkin gene cause autosomal recessive juvenile parkinsonism. Nature.

[2] Valente E.M., Abou-Sleiman P.M., Caputo V., Muqit M.M., Harvey K., Gispert S., Ali Z., Del Turco D., Bentivoglio A.R., Healy D.G. (2004). Hereditary early-onset Parkinson’s disease caused by mutations in PINK1. Science.

[3] Betarbet R., Sherer T.B., MacKenzie G., Garcia-Osuna M., Panov A.V., Greenamyre J.T. (2000). Chronic systemic pesticide exposure reproduces features of Parkinson’s disease. Nat. Neurosci..

[4] Mehta N.J., Asmaro K., Hermiz D.J., Njus M.M., Saleh A.H., Beningo K.A., Njus D. (2016). Hypochlorite converts cysteinyl-dopamine into a cytotoxic product: A possible factor in Parkinson’s Disease. Free Radic. Biol. Med..

[5] Genova M.L., Abd-Elsalam N.M., Mahdy E.S.M.E., Bernacchia A., Lucarini M., Pedulli G.F., Lenaz G. (2006). Redox cycling of adrenaline and adrenochrome catalyzed by mitochondrial Complex I. Arch. Biochem. Biophys..

[6] Mosca L., Tempera I., Lendaro E., Di Francesco L., d’Erme M. (2008). Characterization of catechol-thioether-induced apoptosis in human SH-SY5Y neuroblastoma cells. J. Neurosci. Res..

[7] Hauser D.N., Dukes A.A., Mortimer A.D., Hastings T.G. (2013). Dopamine quinone modifies and decreases the abundance of the mitochondrial selenoprotein glutathione peroxidase 4. Free Radic. Biol. Med..

[8] Vauzour D., Pinto J.T., Cooper A.J.L., Spencer J.P.E. (2014). The neurotoxicity of 5-S -cysteinyl-dopamine is mediated by the early activation of ERK1/2 followed by the subsequent activation of ASK1/JNK1/2 pro-apoptotic signaling. Biochem. J..

[9] Burbulla L.F., Song P., Mazzulli J.R., Zampese E., Wong Y.C., Jeon S., Santos D.P., Blanz J., Obermaier C.D., Strojny C. (2017). Dopamine oxidation mediates mitochondrial and lysosomal dysfunction in Parkinson’s disease. Science.

[10] Biosa A., Arduini I., Soriano M.E., Giorgio V., Bernardi P., Bisaglia M., Bubacco L. (2018). Dopamine oxidation products as mitochondrial endotoxins, a potential molecular mechanism for preferential neurodegeneration in Parkinson’s Disease. ACS Chem. Neurosci..

[11] Herrera A., Muñoz P., Steinbusch H.W.M., Segura-Aguilar J. (2017). Are dopamine oxidation metabolites involved in the loss of dopaminergic neurons in the Nigrostriatal system in Parkinson’s Disease?. ACS Chem. Neurosci..

[12] Nixon R.A., Yang D.S. (2011). Autophagy failure in Alzheimer’s disease—Locating the primary defect. Neurobiol. Dis..

[13] Orr M.E., Oddo S. (2013). Autophagic/lysosomal dysfunction in Alzheimer’s Disease. Alzheimers Res. Ther..

[14] Martin D.D.O., Ladha S., Ehrnhoefer D.E., Hayden M.R. (2015). Autophagy in Huntington disease and huntingtin in autophagy. Trends Neurosci..

[15] Lee J.K., Shin J.H., Lee J.E., Choi E.J. (2015). Role of autophagy in the pathogenesis of amyotrophic lateral sclerosis. Biochim. Biophys. Acta.

[16] Wang B., Abraham N., Gao G., Yang Q. (2016). Dysregulation of autophagy and mitochondrial function in Parkinson’s disease. Transl Neurodegener..

[17] Moors T.E., Hoozemans J.J.M., Ingrassia A., Beccari T., Parnetti L., Chartier-Harlin M.C., van de Berg W.D.J. (2017). Therapeutic potential of autophagy enhancing agents in Parkinson’s disease. Mol. Neurodegener..

[18] Uddin M.S., Stachowiak A., Al Mamun A., Tzvetkov N.T., Takeda S., Atanasov A.G., Bergantin L.B., Abdel-Daim M.M., Stankiewicz A.M. (2018). Autophagy and Alzheimer’s disease: From molecular mechanisms to therapeutic implications. Front. Aging Neurosci..

[19] Pircs K., Petri R., Madsen S., Brattas P.L., Vuono R., Ottosson D.R., St-Amour I., Hersbach B.A., Matusiak-Bruckner M., Lundh S.H. (2018). Huntingtin Aggregation Impairs Autophagy, Leading to Argonaute-2 Accumulation and Global MicroRNA Dysregulation. Cell Rep..

[20] Nguyen D.K.H., Thombre R., Wang J. (2018). Autophagy as a common pathway in amyotrophic lateral sclerosis. Neurosci. Lett..

[21] Hetz C., Thielen P., Matus S., Nassif M., Court F., Kiffin R., Martinez G., Cuervo A.M., Brown R.H., Glimcher L.H. (2009). XBP-1 deficiency in the nervous system protects against amyotrophic lateral sclerosis by increasing autophagy. Genes Dev..

[22] Mukhopadhyay S., Panda P.K., Sinha N., Das D.N., Bhutia S.K. (2014). Autophagy and apoptosis: Where do they meet?. Apoptosis.

[23] Marino G., Niso-Santano M., Baehrecke E.H., Kroemer G. (2014). Self-consumption: The interplay of autophagy and apoptosis. Nat. Rev. Mol. Cell Biol..

[24] Sato S., Uchihara T., Fukuda T., Noda S., Kondo H., Saiki S., Komatsu M., Uchiyama Y., Tanaka K., Hattori N. (2018). Loss of autophagy in dopaminergic neurons causes Lewy pathology and motor dysfunction in aged mice. Sci. Rep..

[25] Wong Y.C., Holzbaur E.L. (2014). Optineurin is an autophagy receptor for damaged mitochondria in parkin-mediated mitophagy that is disrupted by an ALS-linked mutation. Proc. Natl. Acad. Sci. USA.

[26] Lazarou M., Sliter D.A., Kane L.A., Sarraf S.A., Wang C., Burman J.L., Sideris D.P., Fogel A.I., Youle R.J. (2015). The ubiquitin kinase PINK1 recruits autophagy receptors to induce mitophagy. Nature.

[27] Murphy K.E., Gysbers A.M., Abbott S.K., Tayebi N., Kim W.S., Sidransky E., Cooper A., Garner B., Halliday G.M. (2014). Reduced glucocerebrosidase is associated with increased α-synuclein in sporadic Parkinson’s disease. Brain.

[28] Magalhaes J., Gegg M.E., Migdalska-Richards A., Doherty M.K., Whitfield P.D., Schapira A.H.V. (2016). Autophagic lysosome reformation dysfunction in glucocerebrosidase deficient cells: Relevance to Parkinson disease. Hum. Mol. Gen..

[29] Manzoni C. (2017). The LRRK2-macroautophagy axis and its relevance to Parkinson’s disease. Biochem. Soc. Trans..

[30] Galluzzi L., Vitale I., Aaronson S.A., Abrams J.M., Adam D., Agostinis P., Alnemri E.S., Altucci L., Amelio I., Andrews D.W. (2018). Molecular mechanisms of cell death: Recommendations of the Nomenclature Committee on Cell Death 2018. Cell Death Differ..

[31] Wirawan E., Vande Walle L., Kersse K., Cornelis S., Claerhout S., Vanoverberghe I., Roelandt R., De Rycke R., Verspurten J., Declercq W. (2010). Caspase-mediated cleavage of Beclin-1 inactivates Beclin-1-induced autophagy and enhances apoptosis by promoting the release of proapoptotic factors from mitochondria. Cell Death Dis..

[32] Kam T.I., Mao X., Park H., Chou S.C., Karuppagounder S.S., Umanah G.E., Yun S.P., Brahmachari S., Panicker N., Chen R. (2018). Poly(ADP-ribose) drives pathologic α-synuclein neurodegeneration in Parkinson’s disease. Science.

[33] Martire S., Mosca L., d’Erme M. (2015). PARP-1 involvement in neurodegeneration: A focus on Alzheimer’s and Parkinson’s diseases. Mech. Ageing Dev..

[34] Mandir A.S., Przedborski S., Jackson-Lewis V., Wang Z.Q., Simbulan-Rosenthal C.M., Smulson M.E., Hoffman B.E., Guastella D.B., Dawson V.L., Dawson T.M. (1999). Poly(ADP-ribose) polymerase activation mediates 1-methyl-4-phenyl-1, 2,3,6-tetrahydropyridine (MPTP)-induced parkinsonism. Proc. Natl. Acad. Sci. USA.

[35] Kim T.W., Cho H.M., Choi S.Y., Suguira Y., Hayasaka T., Setou M., Koh H.C., Mi Hwang E., Park J.Y., Kang S.J. (2013). (ADP-ribose) polymerase 1 and AMP-activated protein kinase mediate progressive dopaminergic neuronal degeneration in a mouse model of Parkinson’s disease. Cell Death Dis..

[36] Mehta N.J. (2017). Understanding the mechanism of oxidative stress generation by dopamine oxidized metabolites: Implications in Parkinson’s disease. Ph.D. Thesis.

[37] Keane P.C., Kurzawa M., Blain P.G., Morris C.M. (2011). Mitochondrial dysfunction in Parkinson’s disease. Parkinsons Dis..

[38] Johri A., Beal M.F. (2012). Mitochondrial dysfunction in neurodegenerative diseases. J. Pharmacol. Exp. Ther..

[39] Hroudová J., Singh N., Fišar Z. (2014). Mitochondrial dysfunctions in neurodegenerative diseases: Relevance to Alzheimer’s Disease. BioMed Res. Int..

[40] Golpich M., Amini E., Mohamed Z., Ali R.A., Ibrahim N.M., Ahmadiani A. (2017). Mitochondrial dysfunction and biogenesis in neurodegenerative diseases: Pathogenesis and Treatment. CNS Neurosci. Ther..

[41] Murphy M.P. (2009). How mitochondria produce reactive oxygen species. Biochem. J..

[42] Schapira A.H., Cooper J.M., Dexter D., Clark J.B., Jenner P., Marsden C.D. (1990). Mitochondrial complex I deficiency in Parkinson’s disease. J. Neurochem..

[43] Parker W.D., Parks J.K., Swerdlow R.H. (2008). Complex I deficiency in Parkinson’s disease frontal cortex. Brain Res..

[44] Fato R., Bergamini C., Bortolus M., Maniero A.L., Leoni S., Ohnishi T., Lenaz G. (2009). Differential effects of mitochondrial Complex I inhibitors on production of reactive oxygen species. Biochim. Biophys. Acta.

[45] Jin S.M., Lazarou M., Wang C., Kane L.A., Narendra D.P., Youle R.J. (2010). Mitochondrial membrane potential regulates PINK1 import and proteolytic destabilization by PARL. J. Cell Biol..

[46] Greene A.W., Grenier K., Aguileta M.A., Muise S., Farazifard R., Haque M.E., McBride H.M., Park D.S., Fon E.A. (2012). Mitochondrial processing peptidase regulates PINK1 processing, import and Parkin recruitment. EMBO Rep..

[47] Yamano K., Youle R.J. (2013). PINK1 is degraded through the N-end rule pathway. Autophagy.

[48] Narendra D.P., Jin S.M., Tanaka A., Suen D.F., Gautier C.A., Shen J., Cookson M.R., Youle R.J. (2010). PINK1 is selectively stabilized on impaired mitochondria to activate Parkin. PLoS Biol..

[49] Kane L.A., Lazarou M., Fogel A.I., Li Y., Yamano K., Sarraf S.A., Banerjee S., Youle R.J. (2014). PINK1 phosphorylates ubiquitin to activate Parkin E3 ubiquitin ligase activity. J. Cell Biol..

[50] Kondapalli C., Kazlauskaite A., Zhang N., Woodroof H.I., Campbell D.G., Gourlay R., Burchell L., Walden H., Macartney T.J., Deak M. (2012). PINK1 is activated by mitochondrial membrane potential depolarization and stimulates Parkin E3 ligase activity by phosphorylating Serine 65. Open Biol..

[51] Koyano F., Okatsu K., Kosako H., Tamura Y., Go E., Kimura M., Kimura Y., Tsuchiya H., Yoshihara H., Hirokawa T. (2014). Ubiquitin is phosphorylated by PINK1 to activate parkin. Nature.

[52] Thomas K.J., McCoy M.K., Blackinton J., Beilina A., van der Brug M., Sandebring A., Miller D., Maric D., Cedazo-Minguez A., Cookson M.R. (2011). DJ-1 acts in parallel to the PINK1/parkin pathway to control mitochondrial function and autophagy. Hum. Mol. Gen..

[53] Nixon R.A., Wegiel J., Kumar A., Yu W.H., Peterhoff C., Cataldo A., Cuervo A.M. (2005). Extensive involvement of autophagy in Alzheimer disease: An immuno-electron microscopy study. J. Neuropathol. Exp. Neurol..

[54] Lee J.H., Yu W.H., Kumar A., Lee S., Mohan P.S., Peterhoff C.M., Wolfe D.M., Martinez-Vicente M., Massey A.C., Sovak G. (2010). Lysosomal proteolysis and autophagy require presenilin 1 and are disrupted by Alzheimer-related PS1 mutations. Cell.

[55] Martinez-Vicente M., Talloczy Z., Wong E., Tang G., Koga H., Kaushik S., de Vries R., Arias E., Harris S., Sulzer D. (2010). Cargo recognition failure is responsible for inefficient autophagy in Huntington’s disease. Nat. Neurosci..

[56] Maruyama H., Morino H., Ito H., Izumi Y., Kato H., Watanabe Y., Kinoshita Y., Kamada M., Nodera H., Suzuki H. (2010). Mutations of optineurin in amyotrophic lateral sclerosis. Nature.

[57] Majcher V., Goode A., James V., Layfield R. (2015). Autophagy receptor defects and ALS-FTLD. Mol. Cell Neurosci..

[58] Georgakopoulos N.D., Wells G., Campanella M. (2017). The pharmacological regulation of cellular mitophagy. Nat. Chem. Biol..

[59] Chang C.Y., Choi D.K., Lee D.K., Hong Y.J., Park E.J. (2013). Resveratrol confers protection against rotenone-induced neurotoxicity by modulating myeloperoxidase levels in glial cells. PLoS ONE.

[60] Jinsmaa Y., Sharabi Y., Sullivan P., Isonaka R., Goldstein D.S. (2018). 3,4-Dihydroxyphenyl acetaldehyde-induced protein modifications and their mitigation by N-acetylcysteine. J. Pharmacol. Exp. Ther..

[61] Fitzmaurice A.G., Rhodes S.L., Lulla A., Murphy N.P., Lam H.A., O’Donnell K.C., Barnhill L., Casida J.E., Cockburn M., Sagasti A. (2013). Aldehyde dehydrogenase inhibition as a pathogenic mechanism in Parkinson disease. Proc. Natl. Acad. Sci. USA.

[62] Rosengren E., Linder-Eliasson E., Carlsson A. (1985). Detection of 5-S-cysteinyldopamine in human brain. J. Neural Transm..

[63] Shen X.M., Dryhurst G. (1996). Further insights into the influence of L-cysteine on the oxidation chemistry of dopamine: Reaction pathways of potential relevance to Parkinson’s Disease. Chem. Res. Toxicol..

[64] Zucca F.A., Segura-Aguilar J., Ferrari E., Munoz P., Paris I., Sulzer D., Sarna T., Casella L., Zecca L. (2017). Interactions of iron, dopamine, and neuromelanin pathways in brain aging and Parkinson’s disease. Prog. Neurobiol..

[65] Gellhaar S., Sunnemark D., Eriksson H., Olson L., Galter D. (2017). Myeloperoxidase-immunoreactive cells are significantly increased in brain areas affected by neurodegeneration in Parkinson’s and Alzheimer’s disease. Cell Tissue Res..

[66] Scudamore O., Ciossek T. (2018). Increased oxidative stress exacerbates alpha-Synuclein aggregation in vivo. J. Neuropath. Exp. Neurol..

[67] Xiang W., Schlachetzki J.C.M., Helling S., Bussmann J.C., Berlinghof M., Schäffer T.E., Marcus K., Winkler J., Klucken J., Becker C.-M. (2013). Oxidative stress-induced posttranslational modifications of alpha-synuclein: Specific modification of alpha-synuclein by 4-hydroxy-2-nonenal increases dopaminergic toxicity. Mol. Cell. Neurosci..

